# Love Wave Sensor for Prostate-Specific Membrane Antigen Detection Based on Hydrophilic Molecularly-Imprinted Polymer

**DOI:** 10.3390/polym10050563

**Published:** 2018-05-22

**Authors:** Pingping Tang, Yaobin Wang, Jichuan Huo, Xiaoyan Lin

**Affiliations:** 1 School of Materials Science and Engineering, Southwest University of Science and Technology, Mianyang 621010, China; linxiaoyan@swust.edu.cn; 2 School of Computer Science and Technology, Southwest University of Science and Technology, Mianyang 621010, China; 3 Analytical and Testing Centre, Southwest University of Science and Technology, Mianyang 621010, China; huojichuan@swust.edu.cn

**Keywords:** Love wave sensor, prostate-specific membrane antigen, hydrophilic molecularly imprinted polymer

## Abstract

Prostate-specific membrane antigen (PSMA) is a biomarker for prostate cancer (PCa), and a specific and reliable detection technique of PSMA is urgently required for PCa early diagnosis. A Love wave sensor has been widely studied for real-time sensing and highly sensitive applications, but the sensing unit needs special handling for selective detection purpose. In this study, we prepared a versatile Love wave sensor functionalized with molecularly-imprinted polymers (MIP), PSMA as the template molecule. To enhance the specific template bindings of MIP in pure aqueous solutions, facile reversible addition/fragmentation chain transfer (RAFT) precipitation polymerization (RAFTPP) was used to produce surface hydrophilic polymer brushes on MIP. The presence of hydrophilic polymer brushes on MIP improved its surface hydrophilicity and significantly reduced their hydrophobic interactions with template molecules in pure aqueous media. In detection process, the acoustic delay-line is confederative to a microfluidic chip and inserted in an oscillation loop. The real-time resonance frequency of the MIP-based Love wave sensor to different concentrations of PSMA was investigated. The limit of detection (LOD) for this Love SAW sensor was 0.013 ng mL^−1^, which demonstrates that this sensor has outstanding performance in terms of the level of detection.

## 1. Introduction

Prostate cancer (PCa) is one of the most common cancers in men. Early detection of PCa mainly depends on serum prostate-specific antigen (PSA) testing, which is a valuable tool in the staging and monitoring of PCa. Nonetheless, the main disadvantage of PSA testing is its lack of specificity which can result in a high negative biopsy rate. Moreover, PSA is not a PCa-specific event because raising PSA levels can also be detected in men with benign prostatic hyperplasia. Therefore, a specific and reliable detection technique is required for supplementing PCa screening with other cancer markers such as prostate-specific membrane antigen (PSMA).

PSMA is a 100 kDa type II transmembrane protein, with folate hydrolase and glutamate carboxypeptidase II activity. Banerjee et al. found that PSMA is a fairly specific and highly sensitive cancer marker for PCa [1]. Therefore, detecting PSMA may provide a reliable option and first-line procedure for the control of PCa. However, quantitatively detecting of PSMA by traditional methods as western blot or enzyme-linked immunosorbent assay (ELISA) has some disadvantages like time-consuming and severe matrix interference, or the accuracy is not satisfactory. 

Label-free detection methods allow direct, rapid, and sensitive detection of different target analyte in multiplex samples, including cancer markers. For example, Li et al. manufactured graphene–cobalt hexacyanoferrate nanocomposite modified glassy carbon electrode for label-free electrochemical immune-sensing of PSA [2]. Cui et al. established a voltammetric method using Au/TiO_2_ nanobelt hetero-structure electrodes for the detection of lung and breast cancer cells [3]. However, some label-free techniques have not been successfully applied in the reliable detecting of biomarkers in actual samples due to the lack of specificity and sensitivity.

Recently, surface acoustic wave (SAW) sensors for bio-sensing and bio-analysis have been widely studied because of their obvious advantages, such as high sensitivity, capability, and reliability to respond to various analyte [4]. For biological sensing in aqueous samples, shear horizontal (SH) SAW devices are extensively applied, because their dominant in-phase displacement parallel to the substrate provides a minimal damping of the wave in aqueous solution. Furthermore, the SH-SAW can be converted to a Love mode if the wave-guiding layer is coated on top of piezoelectric materials [5]. For the wave-guiding effect, most of the wave energy is limited to this wave-guiding layer, so any small perturbation on the surface could remarkably influence the wave propagation. Consequently, the Love SAW sensors usually have high sensitivity in liquid, which is suitable for bio-sensing applications. In order to ensure specific recognition for target cancer markers, proteins are typically used as recognition biomolecules attached on the sensor surface, using various attachment techniques. However, these natural biomolecules are difficult to produce, expensive, or with limited lifetime and applicability.

Molecularly-imprinted polymer (MIP) is developed to overcome these drawbacks with desirable recognition mechanisms, excellent stability, and high selectivity for target molecules. MIPs offer the possibility of using such materials for a wide range of applications, such as sensing [6], separation [7], and cleanup [8]. For example, Robert et al. explored phospholipid imprinting as an approach to design receptors for complex glycolipids involving the toxic lipopolysaccharide endotoxin [9]. However, most MIPs are often prepared and used in organic environments [10]. This drawback significantly limits the detection of biomarkers in aqueous samples. Up to now, various methods have been attempted to prepare MIPs for the detection of analyte in water. For example, utilizing glycerol dimethacrylate and glycerol monomethacrylate as hydrophilic monomers can synthesize hydrophilic media for the detection of analyte in aqueous media [11]. Qiao et al. prepared water-compatible MIP by water/oil/water suspension polymerization [12]. Zhao et al. designed a facile and highly efficient approach to obtain narrowly dispersed hydrophilic and magnetic MIP microspheres [13]. However, the above methods more or less have some disadvantages, such as cumbersome multi-step graft or swelling step, requiring nitrogen or lower yield. Recently, an effective one-pot technique was used to obtain water compatible MIP with surface-grated hydrophilic polymer brushes by simplistic reversible addition/fragmentation chain transfer (RAFT) precipitation polymerization (RAFTPP), mediated by hydrophilic macromolecular chain-transfer agent (macro-CTA) [14]. The presence of hydrophilic polymer brushes on MIP can obviously improve the surface hydrophilicity and significantly reduce the hydrophobic interactions with template molecules in aqueous solution. The easy availability of different hydrophilic Macro-CTAs, and the versatility of RAFTPP for the controlled preparation of MIP, makes this method highly appropriate for the design of hydrophilic MIP for cancer markers [15].

In the present study, a Love SAW sensor based on water-compatible MIP for the detection of PSMA is fabricated for the first time. In the MIP synthesis process, hydrophilic acylamide (AA), and 2-(dimethylamino) ethyl methacrylate (DMAEM) were used as the functional monomers. Macro-CTA poly hydroxyethyl methacrylate (PHEMA) and normal cumyl dithiobenzoate (CDB) were used as the RAFT agents. The synthesized MIP with hydrophilic polymer brushes on the surface can significantly improve the surface hydrophilicity and enhance the sensitivity and selectivity of the fabricated Love SAW sensor [16]. This novel sensor provided a simple and reliable technique for PSMA detection in biological samples and as a concept to perfect the MIP-based sensor for trace detection.

## 2. Materials and Methods 

### 2.1. Agents and Instruments

PSMA, non-low-density lipoprotein (NLD2), transferrin receptor (TfR1), and paramyosin was purchased from Shanghai Haoran Biological Technology Co., Ltd., Shanghai, China. Functional monomer AA and DMAEM, cross-linker ethylene glycol dimethylacrylate (EGDMA) were all purchased from AlfaAesar, Ward Hill, MA, USA. PSMA-antibodies (200 μg mL^−1^) were from Cell Signaling, Shanghai, China. The PSMA ELISA kit was from Shanghai RenJie Biotechnology Co., Ltd., Shanghai, China. Macro-CTA PHEMA (approximate MW 4800) and normal CDB were purchased from Suzhou Nord Parson’s Pharmaceutical Technology, Suzhou, China. All solutions were prepared with deionized water (>18 MΩ cm). All the other chemicals were of analytical grade. All experiments were carried out at room temperature unless stated otherwise.

To generate a stable flow while remaining extremely reactive, we used a novel flow controller OB1 MkII of Elveflow^®^ (Paris, France) that can control four channels autonomously for a wide variety of innovative microfluidic applications. Each pressure outlet was independently set in a range from 0 to 200 mbar. The liquids are then pressurized into flasks with OB1 MkII pressure controller. Pressurized liquids are smoothly and precisely injected onto the PDMS microfluidic chip, consistent with the set pressure or flow rate profile. Moreover, a switch was placed before the PDMS mircofluidic chip to select the detection liquid.

### 2.2. Fabrication and Pretreatment of Love SAW Sensing Unit

A dual-channel Love Wave sensor consists of a working channel and a reference channel was used in this study. The 20/200 nm thick Ti/Au interdigitated transducers (IDTs) are deposited onto the piezoelectric quartz substrate (ST cut) to generate a shear horizontal surface acoustic wave with a wavelength of λ = 28 μm. This determines that the synchronous frequency is around 160 MHz. A 3-μm thick SiO_2_ film was deposited over the IDTs-patterned substrate by plasma enhanced chemical vapor deposition, and then the contact pads for electrical connection are obtained by etching technology [17].

Then, the Love wave substrate was cleaned by piranha solution (1:1 *v/v*, concentrated sulfuric acid/30% hydrogen peroxide) to eliminate organic and metallic impurities, and establish an oxidation layer on the sensor chip surface. Then, the substrate was rinsed thoroughly with deionized water and dried under nitrogen. Next, it was rinsed with toluene, purged overnight in a silane/toluene mixture, and finally placed in a laboratory oven at 200 °C for 1 h. This silanization process can promote covalent attachment of the MIP layer to the sensing unit. 

### 2.3. Preparation of PSMA Pre-Polymerization Solution

A total of 50 mg template PSMA, 0.1 mmol functional monomer AA, 0.1 mmol DMAEM, and 2.0 mL cross-linker EGDMA were added into a 100 mL methanol/water (4:1 *v/v*) mixture and stirred for 1 h under room temperature. Then, 164 mg macro-CTA PHEMA and 15 mg normal RAFT reagent CDB was added into the above mixture and stirred for another 1 h. The mixture was purged with nitrogen gas for 5 min to remove oxygen. Next, 20 mg initiator azo-bis isobutyronitrile (AIBN) was added, and the flask was sealed with parafilm before mixing for 1 h. The obtained solution was stored in a stained container, for it is light and heat sensitive. A non-imprinted polymer (NIP) pre-polymerization solution was prepared similarly just without the template, for reference purposes.

### 2.4. Thin Film MIP Coating on the Love SAW Sensing Unit 

A 20 μL PSMA pre-polymerization solution was spin-coated on the Love SAW sensing unit. The spin-coating parameters are crucial for the control of the MIP thickness and homogeneity. Typically, acceleration values of 4000 rpm s^−1^ and a velocity of 1000 rpm for 5 s were considered for 200 nm layer thickness. The coating sensing unit was then polymerized under UV light at 365 nm for 1.5 h in a polymerization box with a constant flow of nitrogen. Finally, it was soaked into a 200 mL eluting solution containing ammonium and methanol (70:30 *v/v*, 100 mM aqueous NH_3_/methanol), and stirred overnight. The device was then successively rinsed with ultrapure water (five times) and methanol, and stored in a refrigerator at 4 °C until use. The conversion of the monomers was calculated as 67%.

### 2.5. Detection of PSMA and Regeneration Process

Before the measurement, the prepared MIP-based chip was fabricated on the detection platform. The sensor was stabilized by running PBS (pH = 7.5) for about 30 min. Then the sample solution (500 µL), containing PSMA in the same buffer or mouse serum, was pressurized and precisely injected onto the microfluidic chip, consistent with the set pressure or flow rate profile. The real-time frequency variation of the acoustic wave oscillator was automatically recorded. After detection, the chip was washed with 10 mL eluting solution containing ammonium and methanol (70:30 *v/v*, 100 mM aqueous NH_3_/methanol) to wash the adsorbed PSMA and regenerate the MIP film.

## 3. Results

### 3.1. Scheme of the Chemical Structure of the MIP Film and Hydrophilicity Characterization

**Scheme 1:**
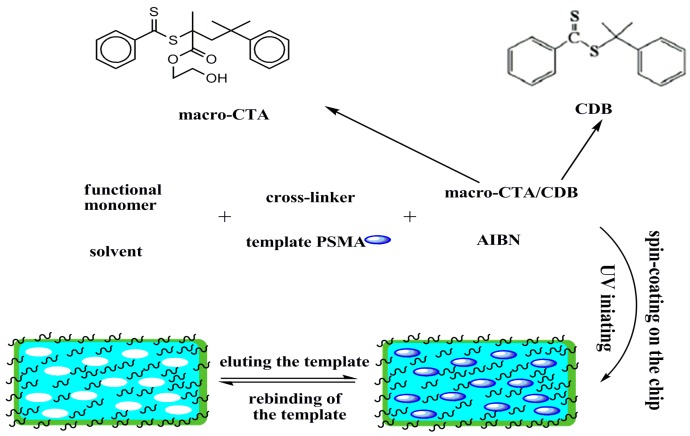
Chemical structures of the utilized RAFT reagents and the schematic procedure for the one-pot preparation of hydrophilic MIP by RAFTPP.

The RAFTPP strategy has been proved to be highly attractive because it not only improves the hydrophilicity of the MIP surface, but also provides a protective layer to prevent protein molecules from blocking their imprinting cavities in biological samples [18]. As shown in Scheme 1, in the MIP synthesis procedure, PSMA was used as the template molecules, EGDMA as the functional monomer, AIBN as the initiator and the mixture of methanol and water (4:1 *v/v*) as the porogenic solvent. Additionally, RAFTPP was carried out in the presence of appropriate macro-CTAs PHEMMA and normal RAFT reagent CDB, because the use of only macro-CTAs leads to irregular MIP surface configuration. In this system, all the reactants were consistent with both the RAFT polymerization and molecular imprinting process.

**Figure 1:**
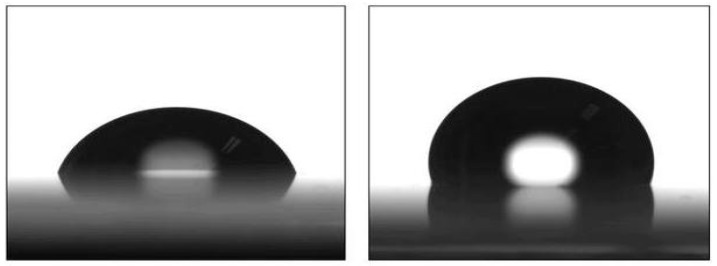
The profile of a water drop on the MIP film with macro-CTAs (**left**) and without CTAs (**right**).

For characterization of the hydrophilicity of the MIP coating sensing unit, the water contact angle test was measured (Figure 1). The contact angle of the MIP film with macro-CTAs was 119 ± 1.8, and that without CTAs was 69.5 ± 2.3. This clearly shows that the contact angle of the PSMA-MIP film was less than 90°. However, if macro-CTAs or normal CTAs were not added in the polymerization process, the MIP film is hydrophobic.

### 3.2. Morphology Characterization of the PSMA-MIP Coating

**Figure 2:**
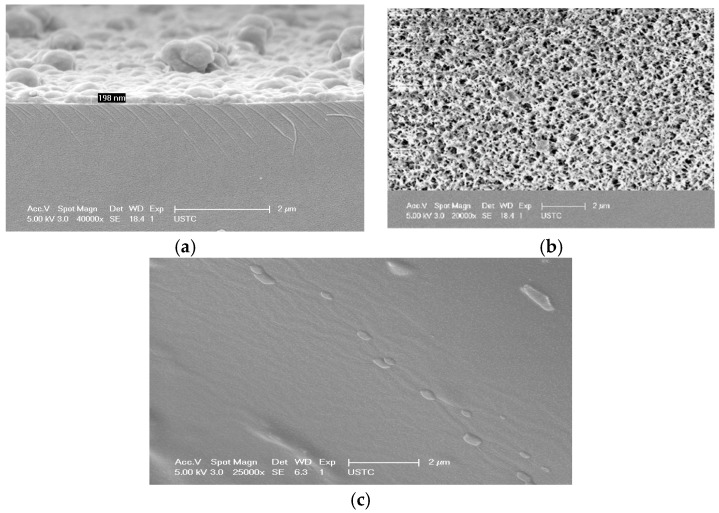
(**a**) the side view SEM of the PSMA-MIP coating sensing unit; (**b**) the top view SEM of the PSMA-MIP coating sensing unit; and (**c**) the top view SEM of the NIP coating sensing unit.

SEM characterization of the so-obtained PSMA-MIP coatings showed typical thicknesses in the range of 100–300 nm, depending on the spin-coater rotation spreading rate. The coating has good surface uniformity and is limited to the wave propagation area of the Love SAW sensor. The thicker the film, the more imprinting sites occurred. However, fast adsorption kinetics is limited because many imprinting sites are wrapped in the MIP network. The thinner the film, the greater the specific surface area is and more imprinting sites are exposed on the surface. Furthermore, the adsorption rate and efficiency can be greatly improved. Thus, in the spin-coating process, values of acceleration 4000 rpm s^−1^ and velocity 1000 rpm for 5 s were considered for about 200 nm layer thickness (Figure 2a). As shown in Figure 2b, the SEM image shows the film surface morphology and the surface is porous. In Figure 2c, the absence of pores in the NIP film can be clearly seen, which demonstrated that there were no imprinting cavities. 

### 3.3. PSMA-MIP Thin Film Function Characterization in Static Mode

**Figure 3:**
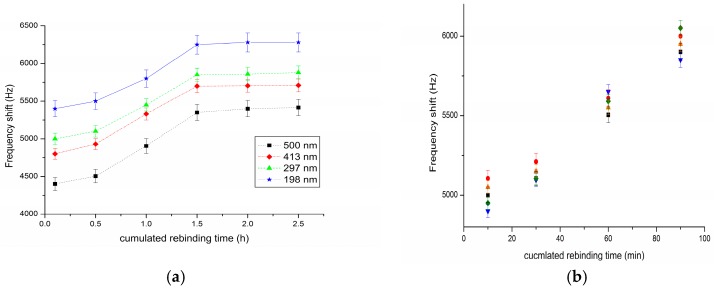
(**a**) Frequency shift (absolute value) of four sensors with different PSMA-MIP film thicknesses to 10 ng mL^−1^ PSMA vs. rebinding time; and (**b**) reproducibility characterization (different colors represent independent sensor by the same fabrication process).

For the detection of PSMA, a concentration of 10 ng mL^−1^ was used to get easily measurable phase and frequency shifts. Such characterizations in static mode allow more insertion losses [19]. Figure 3a describes the absolute frequency shift obtained with four devices with a PSMA-MIP film of different thickness, as a function of cumulated rebinding time, obtained by successive submersion in PSMA solution. It demonstrated that the thinner the film, the larger the frequency shifts. When the rebinding time reaches 1.5 h, the frequency shifts reaches the maximum, which means that the PSMA-MIP film reaches the adsorption saturation. 

For demonstrating the reproducibility of this method, five sensors NO.1–NO.5 with absolutely the same fabrication process were produced and used for the detection of 10 ng mL^−1^ PSMA solution. As shown in Figure 3b, although the devices were functionalized with the MIP film separately, the results indicated quite good reproducibility.

### 3.4. Electrical Characterization 

The MIP-coated sensor was electrically characterized to demonstrate the compatibility of the acoustic propagation with the MIP coating and evaluate the imprinting and coating effect [20]. The coated sensors were inserted in a specific testing cell and the response was recorded in terms of frequency shifts and insertion losses: before and after surface coating, after PSMA extraction from the coating and after rebinding of the template. The 200 nm film coating induced a frequency decrease of about 40 Hz derived from measurements between equiphase points. The MIP-based Love SAW sensor is sensitive to the extraction of PSMA from the MIP film with a frequency shift estimated to about 30 Hz and the PSMA rebinding by the MIP film with a frequency shift of about 2000 Hz for 10 ng mL^−1^ PSMA. Additionally, a PSMA-MIP coating of 198 nm thickness induces about 3 dB insertion losses and no change was noticed after the rebinding step. Considering the mass loading effect, the frequency variations are in accordance with the molecular imprinting principle.

### 3.5. Specificity of the Sensor 

**Figure 4:**
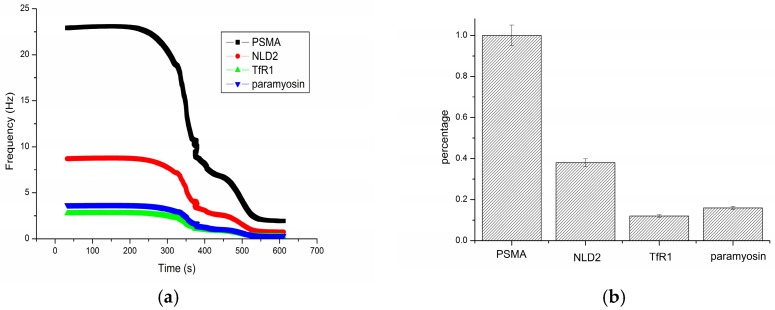
(**a**) Frequency shifts of different proteins; and (**b**) selectivity of the PSMA-MIP based sensor.

In order to investigate the ability of the hydrophilic MIP-based Love wave sensor against interferences arising from the other proteins that are expected to exist in actual samples, selective detection was carried out. NLD2, TfR1 and paramyosin were chosen as the competitive protein. NLD2 and TfR1 were homology proteins of PSMA, and paramyosin has similar molecular weight (100 kDa) of PSMA [21]. The concentration of each protein performed in this specificity testing was 50 ng mL^−1^. The PBS solution without proteins acted as a background group. The response of PSMA was calculated as the difference between PBS and PSMA, and represented as the control group. The response of the other proteins were also calculated as the difference between PBS and the interference species, and represented as percentage from the PSMA response. As shown in Figure 4, PSMA was 1.0 as the control group. NLD2 presented a higher percentage, because it has a similar structure with PSMA. Thus, it can be concluded that the hydrophilic MIP based sensor had a good selectivity for the PSMA detection.

### 3.6. Real-Time Measurements

**Figure 5:**
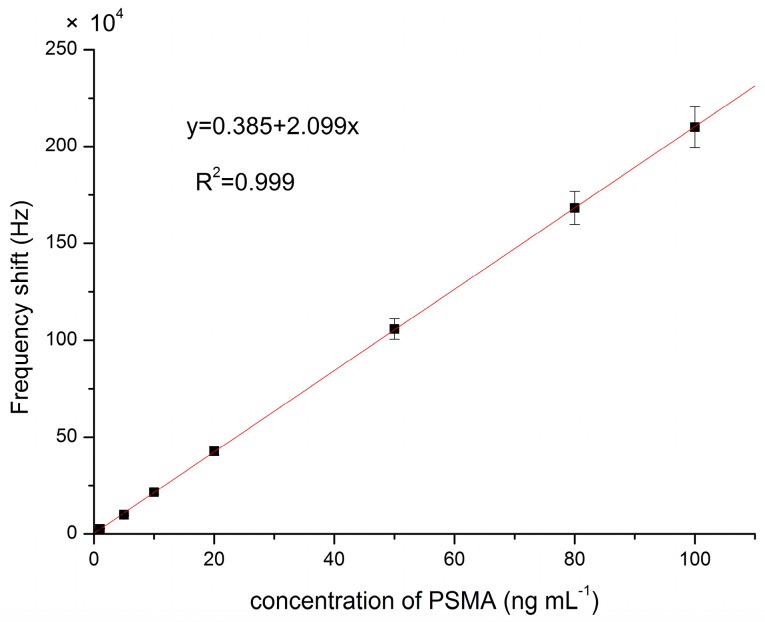
Calibration curve of this method.

In order to characterize the hydrophilic MIP-based sensor response in the presence of target molecules PSMA, the microfluidic chambers were first filled with buffer solution, and then exposed to injection of PSMA of different concentrations. As illustrated in Figure 5, the real time frequency variation of the acoustic wave oscillator coated with PSMA-MIP film was altered to the injection of different concentrations of PSMA.

In the detection of PSMA, the frequency increased with the PSMA concentration and was linearly related to the target concentration in the range of 0.01–100 ng mL^−1^ (Figure 5). The limit of detection (LOD) for this Love SAW sensor was 0.013 ng mL^−1^, and the regression coefficient (*R*^2^) is 0.999. Compared with the other traditional methods reported in the literature, this demonstrates that this sensor has outstanding performance in terms of the level of detection [22,23].

### 3.7. Detection of PSMA in Mouse Serum

Detection of serum cancer markers is an important method for diagnosis and monitoring different cancers due to the ready availability of blood samples. Therefore, we tested our method of the feasibility of PSMA detection by standard addition method in mouse serum. PSMA solutions of various concentration were spiked into serum from mice without tumors and compared to serum alone and spiked PBS as controls. The statistical results are summarized in Table 1. The recovery percentage was varied from 93.8% to 103.9%, indicating that the proposed sensor can be used for the detection of PSMA in relevant samples.

**Figure 6:**
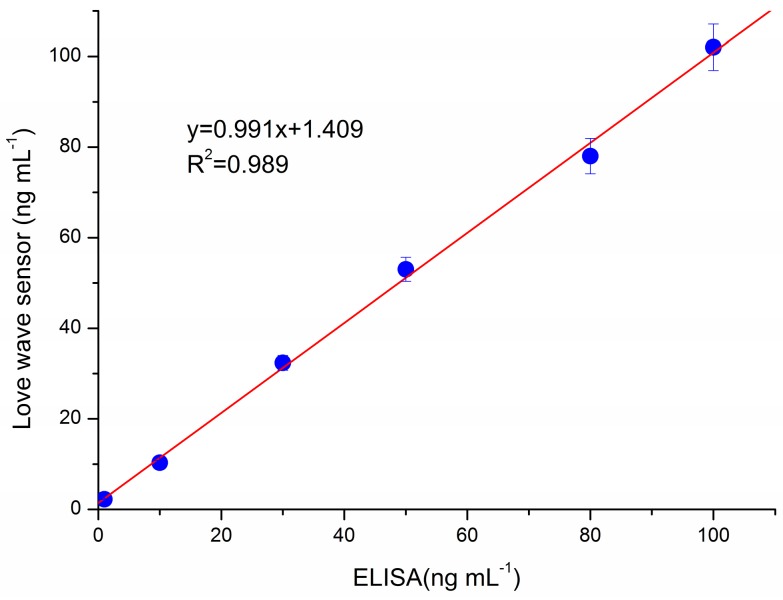
Correlation between the results of the analysis with our Love wave sensor and commercial ELISA assay.

Furthermore, for demonstrating the accuracy of our method in real world sample detection, we compared our method with ELISA assay. Figure 6 shows that the results obtained by the proposed hydrophilic MIP based Love wave sensor were highly correlated with those obtained by the ELISA assay (*R*^2^ = 0.989). However, mouse serum using traditional colorimetric detection methods, such as ELISA, need be diluted by a factor of about 50–1000 to eliminate the background interference [24,25]. Additionally, our method can omit the dilution procedure, which can also save analysis time.

The reusability of MIP materials has a critical role in developing methods that are economic, sustainable, and reliable. The monomers in our method were AA and DMAEM, and the MIP film was regenerated with ammonium and methanol (70:30 *v/v*, 100 mM aqueous NH_3_/methanol) [26]. The experiments were carried out with a 1.0 ng mL^−1^ PSMA solution. The Love wave sensor was regenerated with a frequency shift relative standard deviation (RSD) of 2.93%. Excellent reproducibility was obtained with a RSD of 1.89% after 20 washes and measurements. Moreover, the stability of the sensor was evaluated. The sensor chip was stored in a PBS buffer (pH = 7.5) at room temperature for 10 days, and the frequency shift did not significantly change. After one month, it decreased to 94.15%. All measurements indicated that our sensor possessed excellent reversibility and stability.

## 4. Conclusions

In summary, a Love SAW sensor based on hydrophilic MIP for PSMA detection was described in this study. The MIP was synthesized based on RAFTPP with macro-CTA PHEMA and normal CDB as the RAFT reagent. The contact angle of the MIP film was 119 ± 1.8, which demonstrated its hydrophilicity. In addition, this MIP film had good selectivity between PSMA and its analogues, such as NLD2, TfR1, and paramyosin. In the detection process, the phase shift of the Love wave sensor was linear with respect to the PSMA concentration, in the range of 0.01–100 ng mL^−1^, with a detection limit of 0.013 ng mL^−1^. Compared to other traditional methods reported in the literature, it demonstrates that this sensor has outstanding performance in terms of the level of detection [22,23]. Furthermore, measurement results of the present system have a satisfactory agreement with those of commercial ELISA assays, which confirmed the accuracy of this sensor. Moreover, the frequency shift did not significantly change after 20 washes and measurements or 10 days of storage, which demonstrated the reversibility and stability of our method.

Using the system, different hydrophilic MIP can be synthesized and fabricated as the Love wave sensor sensing unit. Therefore, different biosensor arrays could be developed to detect other cancer biomarkers or proteins. Naturally, in future work, integration and automatization of the platform is required to improve the performance and demonstrate its feasibility in complicated matrix detection.

## Figures and Tables

**Table 1:** Results of the detection of PSMA in mouse serum by our hydrophilic MIP-based Love wave sensor.

Sample	PSMA Added (ng mL^−1^)	PSMA Detected (ng mL^−1^)	Recovery (%)
1	1	0.96 ± 0.11	96
2	10	9.54 ± 1.0	95.4
3	20	18.75 ± 1.9	93.8
4	30	31.09 ± 3.0	103.6
5	50	51.96 ± 5.4	103.9
6	60	57.32 ± 6.9	95.5
7	80	82.43 ± 7.3	103
8	100	103.51 ± 9.8	103.5
